# Remarks on Muscle Contraction Mechanism

**DOI:** 10.3390/ijms9050872

**Published:** 2008-05-23

**Authors:** Toshio Mitsui, Hiroyuki Ohshima

**Affiliations:** 1 Osaka University, Japan; 2 Faculty of Pharmaceutical Sciences, Tokyo University of Science, 2641 Yamazaki, Noda, Chiba, 278-8510, Japan; E-Mail: ohshima@rs.noda.tus.ac.jp

**Keywords:** muscle contraction mechanism, theory, difficulty of the power stroke model, polaron-like model, X-ray equatorial Bragg reflections, tension dependence of muscle stiffness, force-velocity relation, energy liberation rate, isometric tension transient, isotonic velocity transient

## Abstract

Muscle contraction mechanism is discussed by reforming the model described in an article by Mitsui (*Adv. Biophys*. **1999**, *36*, 107–158). A simple thermodynamic relationship is presented, which indicates that there is an inconsistency in the power stroke model or the swinging lever model. To avoid this difficulty, a new model is proposed. It is assumed that a myosin head forms a polaron-like complex with about three actin molecules when it attaches to an actin filament and the complex translates along the actin filament producing force. Various experimental data on the muscle contraction are well explained based upon the model.

## 1. Introduction

The contraction of muscles takes place by mutual sliding of a thick (myosin) and a thin (actin) filaments. A. Huxley [1], H. Huxley [2] and Huxley and Simmons [3] proposed that the filament sliding is generated by power stroke of crossbridges, which occurs once during one ATP hydrolysis cycle. There is a recent review article on “Mechanics and models of the myosin motor” by Huxley [4]. The present authors and their colleagues [5, 6, 7], however, derived a simple thermodynamic relationship, showing that there is an inconsistency in the power stroke model. To avoid this difficulty, they proposed a new model from a completely different viewpoint. The model well explains various experimental data. In the present article, it is tried to interpret the basic idea of the model described in [7] in more readable manner with some refinements.

Difficulties in the power stroke model are discussed in Sect. 2. Basic ideas of the new model are explained in Sect. 3. Various experimental data are theoretically reproduced in Sect. 4. Additional comments are given in Sect.5. The obtained results are summarized and discussed in Sect. 6. A list of the parameter values used in calculation can be found in the Appendix.

## 2. Difficulty in the power stroke model

### 2.1 A thermodynamic relationship

Generally the first step to construct a molecular model in material physics is to look for a thermodynamic relationship among parameters to appear in the model and put restrictions on the manner to construct models (e. g., cf. [8]). Let us follow this procedure in the case of muscle contraction.

It is assumed that a myosin head exerts force to an actin filament only when it attached to an actin. The mean force exerted by the myosin head on the actin filament is denoted as *p* and the translation distance of the attached head during one ATP cycle as *D*, Then *pD* gives the work done by the myosin head utilizing the ATP hydrolysis energy, ε_ATP_. Measured macroscopic quantities during muscle contraction are the tension *P*, the contraction velocity *V* and the rate of heat production *H*. The rate of muscle work *W* is *PV* and the rate of energy production is *W* + *H*. The portion of ε_ATP_ used for work is given by ε_ATP_*W*/(*W* + *H*), and therefore, by the first law of thermodynamics

(2-1-1) $$pD={ɛ}_{\text{ATP}}W/(W+H)\text{.}$$

The tension *P* is equal to the sum of the forces generated by the myosin heads in a thin layer having a unit area cross-section and the width of half the sarcomere length. If *N*_hs_ (hs: half sarcomere length) is the total number of myosin heads contained in this layer and *r* the ratio of the number of heads that are simultaneously in the attached state per *N*_hs_, then

(2-1-2) $$p=P/(r{\text{N}}_{\text{hs}})\text{.}$$

or

(2-1-3) $$r=P/(p\;N_{\text{hs}})\text{.}$$

Combining Eqs. 2-1-1 and 2-1-2 gives

(2-1-4) $$D/r={ɛ}_{\text{ATP}}N_{\text{hs}}V/(W+H)\text{.}$$

This is the required thermodynamic relationship

By inserting the values of ε_ATP_, *N*_hs_ and *V*_max_ (*V* under no load) as given in the Appendix and *W* = 0, *H* = 35.2 kW/m^3^ at the tension *P* = 0 at 0°C (cf. Eq. 4-3-1a) into Eq. 2-1-4, we have

(2-1-5) D/r=900  nm, for P=0  at 0°C.

Additional comments on *D*/*r* are given in Sect. 5.1.

### 2.2 Inconsistency in the power-stroke model

According to Geeves and Holmes [9], the longitudinal displacement of a myosin head during one ATP cycle (power stroke or working stroke, *D* in our notation) is approximately 10 nm. Then Eq. 2-1-5 gives

(2-2-1) r=0.011  (power stroke model).

The stiffness of a muscle, which varies depending upon the stress in the muscle, is assumed to be a measure of *r* in the power stroke mode [10]. According to [10], the ratio of the stiffness of the crossbridge at *P* = 0 to that at *P* = *P*_0_ is 0.35 where *P*_0_ is the isometric tension. Therefore, even in case that the value in Eq. 2-2-1 is for free shortening, i. e. for *P* = 0, the ratio *r* at *P* = *P*_0_ becomes

(2-2-2) $$r=0.011/0/35=0.031,\text{ at}\;P=P_{0}\text{ (power stroke model)}\text{.}$$

On the other hand, the value of *r* at the isometric tension *P*_0_ can be obtained by Eq.2-1-3 using the experimental values of *P*_0_ (cf. Appendix) and *p*_0_ (the isometric tension per head). The experimental results of Ishijima *et al*. [11] indicate that *p*_0_ is close to 5.7 pN. We adopt this value in calculation:

(2-2-3) $$p_{0}=5.7\text{ pN}\text{.}$$

Then Eq. 2-1-3 gives

(2-2-4) $$r=0.41,\text{ at}\;P=P_{0}\text{.}$$

X-ray data are favorable to the *r* value in Eq. 2-2-4. By X-ray diffraction study of the equatorial reflections from a sartorius muscle, Matsubara *et al*. [12] estimated that about 80% of myosin heads migrate around the thin filament in isometrically contracting muscle. Higher values for this percentage (about 92~99%) is reported by Yagi *et al*. [13]. Since these percentages are about twice *r* = 41%, it can be speculated that one of two heads of a myosin molecule attaches to an actin and produces force while the other exists in the vicinity of an thin filament as non-attached relief.

It should be noted that the *r* value of 0.41 in Eq. 2-2-4 is obtained by the thermodynamics and the experimental value of *p*_0_. The *r* value 0.031 in Eq. 2-2-2 derived from the assumption of the power stroke model is much smaller than 0.41. This is a distinct inconsistency in the power stroke model, indicating necessity of a new model based upon a completely different viewpoint.

## 3. Basic ideas in the new model

### 3.1 X-ray diffraction studies suggest constant r

X-ray diffraction data suggest that *r* is almost independent of the shortening velocity. Podolsky *et al*. [14], Huxley [15], Huxley and Kress [16] and Yagi *et al*. [13] observed that the intensity ratio of the [1,0] and [1,1] equatorial reflections increases only minimally as the shortening velocity increases, indicating that the total number of myosin heads in the vicinity of the actin filament decreases only slightly. This observation is commonly explained by assuming that weakly attached crossbridges exist in addition to strongly attached crossbridges and the muscle stiffness is determined by the strongly attached crossbridges. Then a question arises how this weakly attached crossbridge is related to the force production mechanism. It seems reasonable to consider that both the strongly attached and weakly attached crossbridges are substantially related to the force production in positive or negative ways. In our model both are counted to calculate the ratio *r*, i. e., it is assumed that, as an approximation, *r* is constant and independent of the shortening velocity, as the X-ray data suggest. Referring to Eq. 2-2-4, now it is assumed that

(3-1-1) r=0.41,  for any P.

It should be noted that we have

(3-1-2) $$p/p_{0}=P/P_{0}$$

by combining the constant *r* assumption of Eq. 3-1-1 with Eq. 2-1-3. This relation means that we can obtain an expression of a quantity as a function of macroscopic parameter *P*/*P*_0_ when we obtain an expression of the quantity as a function of microscopic parameter *p*/*p*_0_. This is very convenient in theoretical treatment.

As mentioned in the preceding section, the ratio of stiffness of the crossbridge at *P* = 0 to that at *P* = *P*_0_ is 0.35 where *P*_0_ is the isometric tension, and this *P*-dependency of stiffness was attributed to the variation of the ratio *r* of the attached myosin heads in the power stroke model [9]. This *P*-dependency will be explained from a different viewpoint in Sect. 4.1.

### 3.2 Traveling distance of myosin heads along actin filament during one ATP hydrolysis cycle in shortening muscle

According to Eqs. 2-1-5 and 3-1-1, the traveling distance of myosin head *D* during one ATP hydrolysis cycle is given by

(3-2-1) D=369  nm for  P=0  at 0°C.

On the other hand, much smaller values of *D* (5 nm or a few times of 5 nm) have been reported for a single myosin head by several authors. Molloy *et al*. [17] studied movement and force generation produced by a single myosin head and found the movement during one ATP hydrolysis cycle to be about 4 nm. Kitamura *et al*. [18] developed a new assay for direct manipulation of S-1 making visualization possible with a fluorescent label. Their results show that a myosin head moves along the actin filament with 5.3 nm steps and often produces displacement of 11 to 30 nm for each ATP hydrolysis. It seems possible that in their experiment, there was a force to press S-1 onto the actin filament with an effect to facilitate the movement, as discussed later in connection with *f*_J_ (cf. Eq. 3-5-2). Now it seems generally believed that a single myosin head moves along an actin filament by about 5 nm step, with one step or a few during one ATP hydrolysis cycle *in vitro*.

In the new model, *D* is about the period of the myosin strand of 5.46 nm or occasionally a few times of it in the case of a single myosin head, while large values of *D* becomes possible by cooperation of myosin heads in shortening muscle. For readers who hardly believe the large value of *D* in shortening muscle, it should be stressed that the value of 369 nm is based upon thermodynamics, the direct experimental result on *p*_0_ and the equatorial X-ray measurements. Originally the large value of *D* comes from large *D*/*r*. In Sect. 5.1, it will be shown that large values of *D*/*r* comparable to 900 nm in Eq. 2-1-5 can be derived from other experimental data.

In experiments by Ramsey and Street [19], intact isolated muscle fibres from the frog were stretched to various lengths and then stimulated. A roughly linear decline of active force with extension of the fibre was observed beyond the length at which it gave maximum (cf. Figure 1 in [4]). From this fact it is commonly concluded that the myosin head produces force independently. This reasoning is, however, based upon the observation in isometric contraction and it seems too speculative to consider that the heads produce force independently also in shortening muscle. There should be some time duration that the myosin filament exerts a force to a myosin head bound to an actin filament in shortening muscle. This force is originally due to other myosin heads. In this sense there is an mutual interaction or cooperation among the heads, as will be discussed in more detail in Sect. 3. 7.

### 3.3 Formation of molecular complex of myosin head and actin molecules

Yagi *et al*. [13] observed that X-ray intensities of the 5.9 and 5.1 nm actin layer lines change depending upon the tension, indicating that molecular deformation occurs during force generation. Borejdo and his colleagues [20, 21] studied the way of binding of a myosin head to an actin filament by using tryptic digestion of myofibrils and measuring optical polarization and dichroism. They concluded that in the rigor rabbit psoas muscle each myosin head binds to two actin monomers in a thin actin filament [21], suggesting the possibility that the myosin head may first bind to one and then to two monomers in F-actin [20].

**Figure 1.**
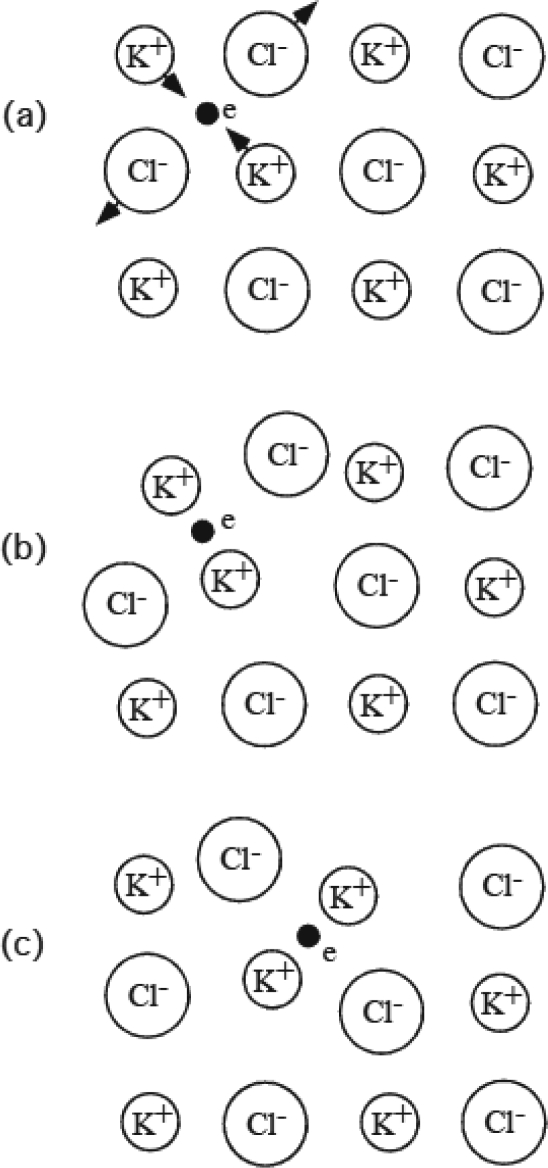
The formation of a polaron in two-dimensional models of ionic crystal, following Figure 19 of [22]. (a) The small filled circle shows electron e in a rigid lattice of an ionic crystal, KCl. The arrows show the forces on the ions adjacent to the electron. (b) Electron e in deformable lattice. The electron plus the associated strain is called polaron. (c) The polaron moves to the next site, changing the mate ions by hopping over a potential barrier.

Generally, when a molecule A bounds to an assembly of B molecules, structural changes occur in both A and B and rearrange the manner of the binding, resulting in formation of the locally deformed complex of A and B. If molecules A and B are electrically charged and have no center of symmetry (i. e. structurally polar) as in the case of protein molecules, the complex formation will be enhanced by electric and piezoelectric interactions. It seems plausible that sometimes the molecular complex moves in the assembly of B as in the case of a polaron in ionic crystals. Presumably some readers are not familiar with the polaron, and a simplified scheme of the polaron formed by an electron in two-dimensional KCl crystal is illustrated in Figure 1, referring to Figure 19 of Kittel's text book [22].

In Figure 1(a), the small filled circle shows electron e which gets into a ligid two-dimensional lattice of ionic crystal, KCl. The arrows show the forces on the ions adjacent to the electron. Figure 1(b) shows the case of a deformable lattice. The adjacent K^+^ ions move closer to e and Cl^−^ ions apart from e, forming the strain field around e. The electron plus the strain field is called a polaron. The polaron moves in the crystal by hopping over a potential barrier, changing the mate ions (c).

**Figure 2.**
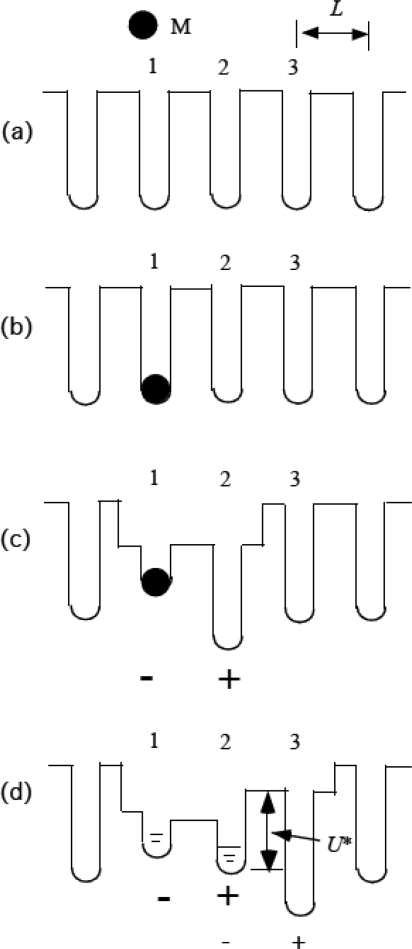
Diagram showing sequential changes in the potential of force acting on ATP-activated single myosin head M (subfragment-1) in binding to an actin filament. The numbers 1, 2, 3 are assigned to the potential wells at the binding site on actin molecules on the same strand. (a) Periodic potential distribution when M is sitting at a position apart from the actin filament. (b) Just after M attaches to actin 1. (c) Molecules deform and potential distribution changes. (d) Equilibrium potential distribution. M is statistically distributed in wells 1 and 2. U* is the potential barrier for M to move to well 3, which is high in the case of single myosin molecule.

The large value of *D* makes us suppose that a molecular complex somewhat similar to a polaron is formed between myosin and actin molecules in muscle. As mentioned above, there is the experimental observation suggesting that a myosin head may first bind to one and then to two monomers in F-actin [20]. Figure 2 shows an example of possible mechanisms of how such a complex of a myosin head and actin molecules is formed in terms of potential distribution for the myosin head. In Figure 2(a), ATP-activated single myosin head (S-1) is indicated as M, which sits at a position apart from the actin filament. The periodic array of potential wells shows the possible binding sites for M along the actin filament with a period of the strand (*L* = 5.46 nm). The helical structure of the actin filament will disturb the periodic potential arrangement but its effect does not seem essential and is neglected here. The myosin head is considered to move to the right during filament sliding and hereafter this direction is called forward and the opposite direction backward. Figure 2(b) shows the state at the moment M attaches to actin 1. As mentioned above, attachment of the myosin head may cause large strain in actin molecules. The actin filament is structurally polar and hence piezoelectric. Consequently, it is possible that the strain produces an electric polarization as symbolically indicated by the electric charges - in actin 1 and + in actin 2 in Figure 2(c). The myosin head is negatively charged and the Coulomb interaction between the head charge and the induced polarization charges raise the potential well at the actin molecule 1 and lowers it at actin 2, resulting in the potential energy distribution for the head shown in Figure 1(c), which might be wide over the two actin molecules and has two narrow inner potential wells at the actin molecule 1 and 2, such that M can jump from one to the other due to the thermal fluctuation and is statistically distributed in these wells as schematically drawn in Figure 2(d). The negative charge of the head will tend to stabilize the charges caused by the polarization in wells 1 and 2. The statistical distribution in the wells was treated based upon statistical mechanics in connection with the isometric tension transients in [7] as outlined in Sect. 4.4. The result in [7] suggests that the probability of the head sitting in the potential well 2 is much larger than that in well 1. The head at well 2 will newly produce the polarization charge – in well 2 and + in well 3 similarly to the case of (c) but somewhat differently due to the difference in prehistory from (c). The induced charges in this case are symbolically shown by small – and + in (d) Accordingly, the structural changes in (d) will be similar to (c) but somewhat different from (c). The potential distribution is supposed to be such as shown in (d) with a potential barrier U*. If this barrier U* is low, the head can move to right. But it is assumed that U* is so high that the head hardly jumps over it to right in the case of a single myosin head. Thus the attached single head usually moves only over the length *L*, i. e. about 5 nm, the generally accepted distance as discussed in Sect. 3.2. In shortening muscle, however, cooperation of the myosin heads decreases U* and realizes the long traveling distance of the head, as will be discussed in the following sections.

Rayment *et al*. [23, 24] proposed that structure of the myosin molecule after force generation is that of rigor of which crystal structure was analyzed. The myosin head consists of a globular catalytic domain that binds to an actin and hydrolyzes ATP and a neck domain that consists of essential and regulatory light chains bound to a longα-helical portion of the heavy chain. The molecular deformation can occur at the junction between the two domains. In the negative stain and single-particle analysis by Burgess *et al*. [25], there is considerable flexibility between the catalytic and neck domains, despite the molecules having been prepared in the absence of nucleotide.

**Figure 3.**
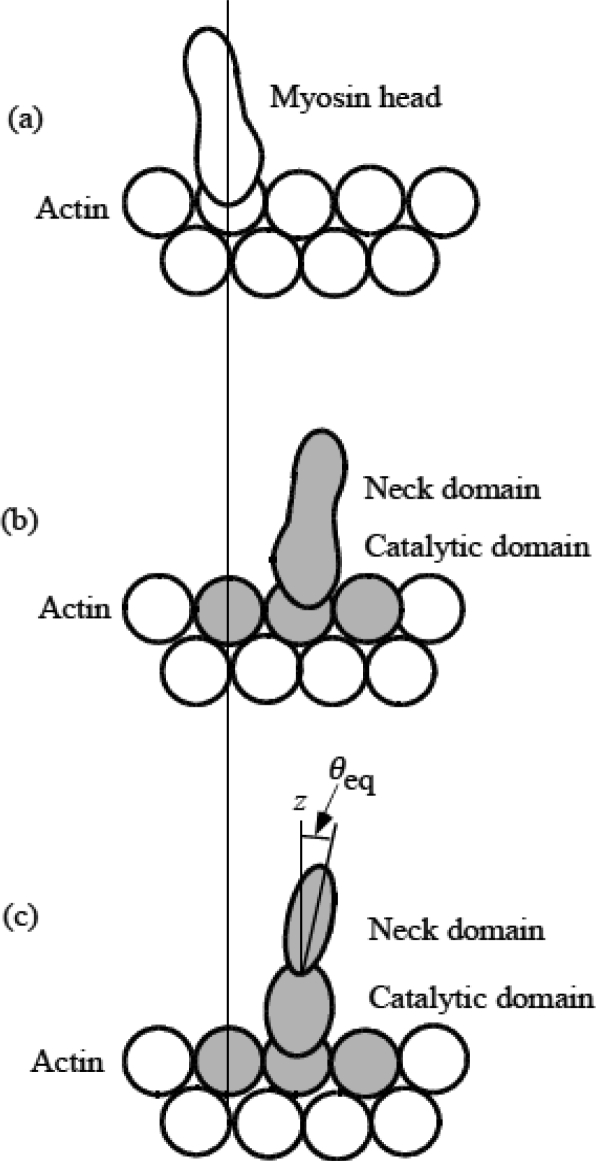
Formation of a complex of myosin head and actin molecules (MA_3_). (a) Just after attachment of myosin head to actin molecule corresponding to Figure 2(b). Molecules are not deformed yet. (b) Formation of MA_3_ corresponding to Figure 2(d). (c) Modeling of (b). The angle θ_eq_ is the bending angle of neck domain at equilibrium.

It is assumed that molecular structures change as shown in Figure 3, associated with the changes in potential distribution in Figure 2. Figure 3(a) shows a myosin head just after the attachment to actin filament (the shape is speculated), which corresponds to the state in Figure 2(b). Then the potential changes occur from Figure 2(b) to (c) and (d), causing the shift of the binding site of the head from Figure 3(a) to (b). The shape of the head in (b) is depicted referring to the shape in Figure 6 of [24]. As in Figure 2(d), it is assumed that the molecular deformation occurs mainly in the three shadowed actin molecules together with the head, and call the shadowed complex in Figure 3(b) MA_3_. Figure 3(c) is a modeling of (b). The tilting angle of the neck domain relative to the vertical *z* axis is denoted as θ_eq_ in the case of a single myosin head.

Figure 2(d) shows that the myosin head exists in wells 1 and 2 although the molecular deformation extends to actin 3. Hence it is plausible that the head binds to myosin 1 and 2 more tightly than 3. This may be related with the observation by Borejdo and his colleagues [20, 21] cited in Sect. 3. 3. They studied the way of binding of a myosin head to an actin filament, concluding that in the rigor rabbit psoas muscle each myosin head binds to two actin monomers in a thin actin filament [21],

### 3.4 Elastic deformation and force production of crossbridge

**Figure 4.**
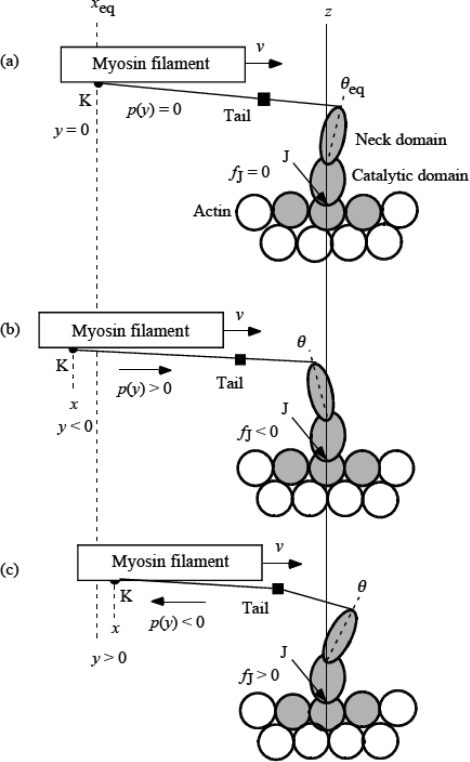
Deformation of a myosin head while the catalytic domain stays at the same actin molecule. The small square on the tail is to show symbolically the bending flexibility of the tail. (a) Myosin head is at the equilibrium angle, θ_eq_. (b) Myosin head is pulling myosin filament forward. (c) Myosin head is pushing myosin filament backward.

Now a contracting muscle is considered. The myosin filament is thought to be moving to the right at a constant velocity *v* with respect to the actin filament in Figure 4. It is assumed that the neck domain can elastically bend against the catalytic domain as shown in Figure 4. The tilt angle θ is defined referring to the vertical *z* axis and is positive for rightward tilt. The equilibrium state in which a myosin head does not produce tension is shown in Figure 4(a), where *θ* is indicated as θ_eq_ assuming that the molecular shape is the same as the single myosin head in Figure 3(c). The restoring force increases with | θ − θ_eq_ |. The stress which the myosin head exerts on the myosin filament is indicated as *p*(*y*) in Figure 4 (*y* will be defined below). *p*(y) is positive when θ − θ_eq_ < 0 (Figure 4(b)) and negative when θ – θ_eq_ > 0 (Figure 4(c)). Wakabayashi and Yagi and their colleagues [26] measured intensity changes of the 14.5 nm meridional reflection by applying sinusoidal length changes (peak-to-peak amplitude 0.6% of the fiber length, 500 Hz) to muscle fibers during active contraction and in rigor. The 14.5 nm meridional reflection sensitively depends on the electron density of a myosin head projected onto the meridian. The density projection profile will become broad and the reflection intensity decreases in release if the mean θ is positive, while the projection will become sharp and the intensity increase in release if the mean θ is negative, for such small oscillation amplitudes as 0.6%. Wakabayashi *et al*. [26] observed that the 14.5 nm intensity changed in phase in rigor and in antiphase in active contraction against the sinusoidal length change, indicating that θ_eq_ is positive and the mean θ in the isometric contraction is negative as shown in Figure 4(a) and (b).

1973, Mendelson *et al*. [27] examined the flexibility of rabbit myosin and heavy meromyosin by the nanosecond fluorescence depolarization technique and found that there is considerable flexibility within heavy meromyosin which is localized near the S1-S2 connecting joint. Elliott and Offer [28] examined myosin molecules of rabbit skeletal muscle by electron microscope, and demonstrated that there is a hinge at the head-tail junction where a myosin molecule can bend flexibly. They also frequently observed that molecules are bent sharply back onto themselves at a well defined position along the myosin tail. Walker *et al*. [29] found another hinge region in the tail and noted that the tail is seldom perfectly straight, all parts of it being sufficiently flexible to allow it to curve gently. Accordingly, the tail will not be stiff for a shortening force, while it will be stiff for expanding force after it is straightened because of itsα-helical structure. Figure 4 illustrated the situation schematically. For simplicity, the bending freedom of the tail is represented by one hinge depicted as a black square. When the head pulls the myosin head (*p*(*y*) > 0, Figure 4(b)), the tail is straightened and the elastic force by the myosin head will be straightforwardly transmitted to the myosin filament. When the head presses the myosin head (*p*(*y*) < 0, Figure 4(c)), there will be some bending in tail and the effect of the elastic force will be weakly transmitted to the myosin filament.

The crossbridge is the tail plus myosin head. In Figure 4, the end of the tail on the myosin filament is indicated as K and the end of catalytic domain on the actin filament as J, so that the crossbridge is the material system from K to J.

The *x* coordinate is defined along the filament axis with positive direction to right putting the origin on the *z* axis. The *z* axis is set on J, and thus *x* of K is negative as seen in Figure 4. The position of K at θ_eq_ is indicated as *x*_eq_ (Figure 4 (a)). The length change of the crossbridge is represented by

(3-4-1) $$y=x\;-\;x_{\text{eq}}\text{.}$$

By definition, *y* is negative in Figure 4(b) and positive in (c), and represents shirinkage of the crossbridge. The force which the crossbridge exerts on the myosin filament is denoted as *p*(*y*). There is a force exerted on J as a reaction to *p*(*y*), which is denoted as *f*_J_:

(3-4-2) $$f_{\text{J}}=-p(y)\text{.}$$

Note that *f*_J_ has the same sign as *y* as seen in Figure 4. It is assumed that Hooke's law approximately holds for *f*_J_. Then,

(3-4-3) $$f_{\text{J}}=\kappa y$$

where κ is the mean stiffness of the crossbridge. Since the head and tail are connected in series as elastic elements, we have

(3-4-4) $$1/\kappa =1/\kappa_{\text{H}}+1/\kappa_{\text{T}}\text{.}$$

where κ_H_ and κ _T_ are the stiffnesses of the myosin head and tail, respectively. When *p*(*y*) is positive (Figure 4(b)), the stiffness κ_T_ seems much larger than κ_H_ due to the α-helical structure of the tail, and κ is nearly equal to κ_H_. On the other hand, when *p*(*y*) is negative (Figure 4(c)), κ_T_ should be much smaller than κ_H_ due to the bending flexibility of the tail and κ is nearly equal to κ_T_. Thus, if κ is expressed by κ_f_ for the forward force (*p*(*y*) > 0) and by κ_b_ for the backward force (*p*(*y*) < 0), κ_f_ is nearly equal to κ_H_ and κ_b_ is nearly equal to κ_T_, and κ_f_ is expected larger than κ_b_ With these notations, Eq. 3-4-3 becomes

(3-4-5a) $$f_{\text{J}}=\kappa_{\text{f}}y,\text{ for}\;y<0,$$

(3-4-5b) $$f_{\text{J}}=\kappa_{\text{b}}y,\text{ for}\;y>0.$$

Then, from Eq. 3-4-2,

(3-4-6a) $$p(y)=-\kappa_{\text{f}}y,\text{ for}\;y<0,$$

(3-4-6b) $$p(y)=-\kappa_{\text{b}}y,\text{ for}\;y>0.$$

**Figure 5.**
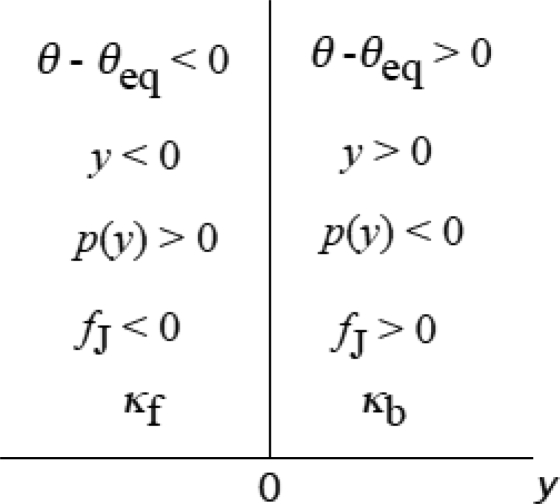
Various quantities in the domain of *y* = *x* − *x*_eq_ (Eq. 3-4-1)

Magnitudes of κ_f,_ and κ_b_ will be determined in Sect. 4. 1. Figure 5 summarizes the quantities mentioned above in the domain of *y*.

### 3.5 Step motion of myosin head along actin filament

**Figure 6.**
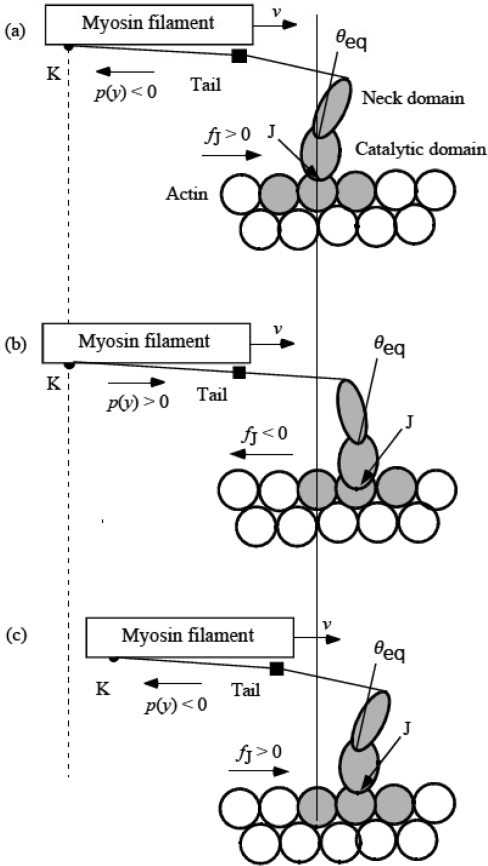
Step motion and deformation of a myosin head in shortening muscle. (a) Rightward tilt of neck domain just before catalytic domain moves to right. (b) Leftward tilt of neck domain just after catalytic domain moves to the new site. (c) Rightward tilt of neck domain when catalytic domain is ready to next movement to right.

Figure 2(d) showed the potential barrier U*. According to Eyring's theory of the rate process [30], the probability that the myosin head moves from well 2 to 3 across the barrier U* is proportional to exp(-U*/*kT*). Figure 2 is drawn for the case of a single myosin head where *f*_J_ = 0 and U* is considered to be so high that the head hardly move to right. In shortening muscle, however, there is the time duration that the myosin head tilts right over θ_eq_ and *f*_J_ becomes positive as shown in Figure 4(c). It is assumed that U* decreases as *f*_J_ increases so that the transition from well 2 to 3 becomes possible. Figure 6 illustrates the transition by the molecular model. Figure 6(a) shows the state where *f*_J_ becomes so large that the catalytic domain is going to translate to the next site. Figure 6(b) shows just after the head translates to a new site. Now θ − θ_eq_ < 0 and the head will pull the myosin filament forward till θ reaches θ_eq._ After θ increases over θ_eq_, the state will become as shown in Figure 6(c), which is the same as Figure 6(a) except for the binding site and the head is ready to translate to right. The myosin head moves along the actin filament by repeating such steps.

Based upon Eyring's theory of the rate process [30], the probability *Q* that the myosin head moves across the barrier U* is given by

(3-5-1) Q=Aexp(-U*/kT),

where *A* is a constant. To express the tendency that U* decreases when *f*_J_ increases for *y* > 0, we consider an approximate expression:

(3-5-2) $$U*=U{*}_{0}-af_{\text{J}},$$

where *a* is a constant. Since *y* is positive under consideration, *f*_J_ is given by κ_b_ *y* (Eq 3-4-5b). With a parameter *b* defined by

(3-5-3) $$b=a\kappa_{\text{b}},$$

Eq. 3-5-2 becomes

(3-5-4) $$U*=U{*}_{0}-\;by\text{.}$$

Hence *Q* is proportional to exp(*by*/*kT*). Since exp(*by*/*kT*) increases rapidly with increasing *y*, most transitions will occur around some *y* which is denoted as *y*_c_ (c: critical). Hereafter, we suppose that all transitions occur simultaneously when *y* reaches *y*_c_. This approximate treatment will make various calculations simple.

### 3.6 Cycles of force generation and the isometric tension

**Figure 7.**
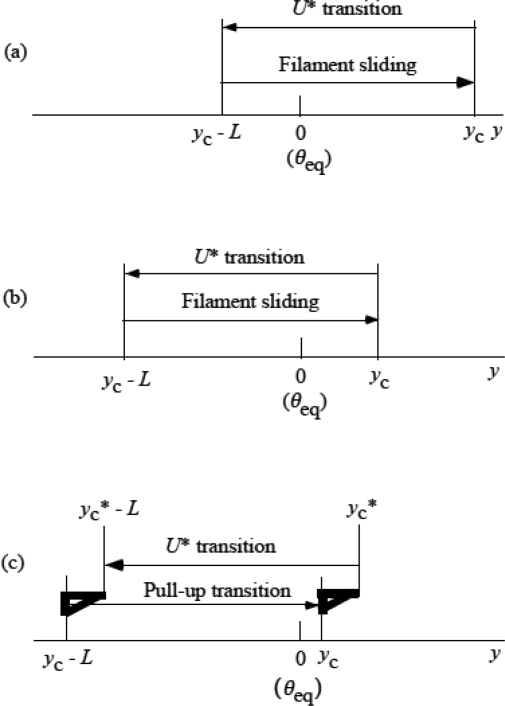
Cycle of variation of *y*. (a) Fast sliding. (b) Slow sliding. (c) Near the isometric tension, *P*/*P*_0_ > 0.68.

Figure 7 illustrates our idea on muscle contraction in the domain of the variable *y*. Let us call the translation of the head over U* “U* transition”. The head in Figure 6(a) is just before U* transition and its *y* is *y*_c_ defined in the preceding section. The head in Figure 6(b) is just after U* transition and its *y* is *y*_c_ − *L* as J shifted by *L*, by definition of *y* (Eq. 3-4-1). Thus U* transition is associated with *y* change from *y*_c_ to *y*_c_ − *L* as indicated by the arrow “U* transition” in Figure 7(a) and (b). The head in Figure 6(b) is also at the starting point to produce the force. The head in Figure 6(c) is just before U* transition and its *y* is *y*_c_. Thus *y* changes from *y*_c_ − *L* to *y*_c_ with the filament sliding in the process from Figure 6 (b) to (c)This process is indicated by the arrow “Filament sliding” in Figure 7(a) and (b). The myosin head produces the force during this “Filament sliding”. When the filament sliding is fast, the transition probability *Q* =*A*exp(−U*/*kT*) (Eq. 3-5-1) should be large, and thus U* is small and *y* is large according to Eq. 3-5-4. Therefore, *y*_c_ is large for fast sliding as shown in Figure 7(a) and small for slow sliding as in (b).

A question arose of what determines the isometric tension, where the sliding velocity is zero and the above cycle stops. In this connection, the double hyperbolic force-velocity relation found by Edman [31] is interesting. As will be proved in Sect. 4.2, the new model leads us to the so-called hyperbolic force-velocity relation. Edman [31], however, found distinct deviation from the hyperbolic force-velocity relation when the relative stress *P*/*P*_0_ becomes larger than about 0.68 (cf. Figure 11) and called the observed result double hyperbolic force-velocity relation. In [7], this deviation was attributed to sudden increase of U* near *y*_c0_ (*y*_c_ at *P*_0_) but this explanation does not seem very realistic. Instead, another mechanism is proposed here that the isometric tension is related with a tolerance limit for “pull-up” detachment of the head from the potential well 3 toward well 2 in Figure 2(d). That is, a forced transition from the state of Figure 6(b) to that of Figure 6(a) takes place when the stress *P*/*P*_0_ increases beyond 0.68. In Figure 7(c), the thick triangle symbolically shows that this effect starts at *y*_c_ = *y*_c_* which corresponds to *P*/*P*_0_ = 0.68, increasing with decreasing *y*_c_. Then, *y* of some myosin heads will change their binding positions from the region of *y*_c_ − *L* < *y* < *y*_c_* − *L* to *y*_c_ < *y* < *y*_c_* as indicated by the arrow “Pull-up transition”. The U* transition will occur in the opposite direction also for these heads and keep the system stationary. The pull–up reduces the population of the head having large *p*(*y*) in *y*_c_ − *L* < *y* < *y*_c_* − *L* and increases the population having negative *p*(*y*) in *y*_c_ < *y* < y*, as seen in Figure 7(c). This effect increases with decreasing *y*_c_ as the region *y*_c_ < *y* < *y*_c_* is widened, implying that reduction of *P* due to the pull-up will increase with increasing {(*P*/*P*_0_) – 0.68} (cf. Figure 11). It seems plausible that such dynamical processes are proceeding in the isometric tension.

### 3.7 Cooperativity of myosin heads, and energy flow and chemical reactions associated with force production

The potential barrier U* is reduced by the force *f*_J_ as U* = U*_0_ − *a f*_J_ (Eq, 3-5-2), where *f*_J_ is the force exerted on the head by the myosin filament. This force is originally produced by other myosin heads attached to the myosin filament. In this sense, myosin molecules belonging to the same myosin filament help each other to make their step motion easy by decreasing others’U*. Due to this cooperation, the amount of energy used in each step becomes small and the head can travel over the long distance *D*. Values of *D* will be calculated as a function of *P*/*P*_0_ in Sect. 4.3 (cf. Figure 14).

**Figure 8.**
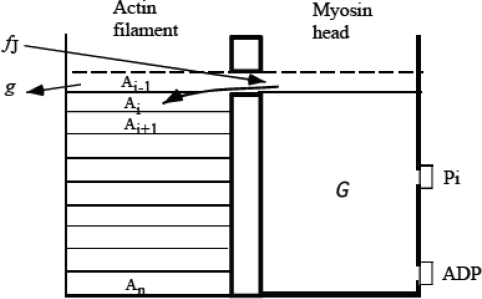
Energy flow and chemical reaction associated with force production. Chemical reaction is a series of detachment from actin A_i−1_ and attachment to A_i_. The fraction, *g*, of the free energy of myosin head is used for force production at each chemical reaction. The energy stored in the head after the ith step is denoted by *G*. The initial value of *G* is ε_ATP_ given to the head by the ATP hydrolysis.

Lymn and Taylor [32] proposed the cycle relating the force production with chemical reactions. In their scheme, dissociations of Pi and ADP play the important role in force production (cf. e. g., [9]). The ways of energy flow and chemical reaction are quite different from those in the new model and dissociations of Pi and ADP do not play important role in connection with force production. Portions of the ATP hydration energy ε_ATP_ stored in the head are used in forming the complex MA_3_ (Figure 3(b)) and the force generation steps. Each step of force generation is associated with the chemical reactions: dissociation from one actin molecule and binding to the neighboring actin molecule. Figure 8 is an illustration of the scheme of the new model. On the right, myosin head is depicted as a box with the energy *G* stored in it. The level of *G* is lowered successively associated with the step of force production of the myosin head. Actin filament is depicted as a box with many shelves on the left. Shelf A_i_ corresponds to ith actin molecule. The force *f*_J_ lowers the potential barrier U* and let the head dissociate from A_i–1_ and bind to A_i_. Associated with this step, myosin head produces force by spending the energy indicated as *g*. Energy liberation rate will be calculated in Sect. 4. 3.

### 3.8 Role of thermal fluctuation

It is assumed that the probability *Q* for myosin head to cross over U* is expressed by *Q* = *A*exp(−U*/*kT*) (Eq. 3-5-1) following Eyring [30]. In usual text books, Eyring's theory of rate process is considered about the probability that a single material particle crosses over a potential barrier U*. Then, the energy for the particle to cross over U* is supplied by the thermal energy of the surroundings of the particle. In the case of a myosin head, however, the head has much internal freedom for structural and thermal fluctuation. Hence it seems plausible that the fluctuation occurs adiabatic in a limited time scale: Decrease of structural fluctuation energy is compensated by increase of thermal fluctuation energy of constituent atoms, and vise versa. Thus the internal energy of the head is used for the head to detach from the actin molecule 2 and attach to actin 3 in Figure 2(d). While the head drops down to the potential well 3, the potential energy of the head will be converted into the elastic energy of the crossbridge to be used to pull the myosin filament. In this way the internal energy of the head originally supplied by ATP hydrolysis is used by parts to pull the myosin filament. The thermal fluctuation plays important role for force production but there is no contradiction against the second law of thermodynamics.

This idea is similar to the model proposed by Huxley in 1957 [1] in the sense that the thermal energy plays an important role to produce force. In Huxley model, however, most of the ATP hydration energy ε_ATP_ (about 21*kT*) is spent at once for one power stroke. Then the probability appears too small for the event to occur. In our model, only a fraction of ε_ATP_ is used in each cycle of force production. The magnitudes of the energy fraction will be discussed in Sect. 4.3.

### 3.9 Isometric tension transient

Huxley and Simmons [3] studied the response of a frog muscle fiber in isometric tension to stepwise length changes. Ford *et al*. [33], who extended the above study, carried out many calculations on their experimental results. Their experimental results were explained differently from the viewpoint of a new model in [6, 7]. In Sect. 4. 4, contents of [7] are outlined citing some of the calculation results in [7].

### 3.10 Isotonic velocity transient

Isotonic velocity transients were studied by Podolsky [34], Civan and Podolsky [35] and Huxley [36]. A muscle was stimulated and initially held at a constant length. It was then released suddenly and allowed to shorten under a constant load. When the muscle length is strictly fixed as in the experiment of the isometric tension transient, the population ratio of the myosin heads in wells 1 and 2 in Figure 2(d) is uniquely determined for the stationary state. On the other hand, when the load is fixed as in the experiment of the isometric velocity transient, the relative populations in wells 1 and 2 are not uniquely defined. Thus the response of muscle becomes complex in isotonic velocity transient, but becomes understandable from viewpoint of the new model [7]. Outline of the discussion in [7] is given in Sect.4.5.

## 4. Quantitative explanation of experimental data

### 4.1 Tension dependence of muscle stiffness

As already mentioned n Sect. 2.2, the stiffness of a muscle varies depending upon stress, and is regarded as a measure of *r* in the power stroke mode [10]. In the new model, *r* is assumed constant and the experimental result is explained as follows.

The X-ray diffraction studies on the extensibility of myosin and actin filaments by Huxley *et al*. [37] and Wakabayashi *et al*. [38] indicated that the total extensibility of these filaments is equal to or more than that of the crossbridges. As discussed by Irving [39], however, the filament would be effectively inextensible during each step motion of an individual myosin head since a few hundred heads simultaneously interact with each actin filament. Accordingly, we shall discuss force generation in the crossbridge mechanism as if extensibility existed only in the crossbridge,

The stress *p*(*y*) produced by myosin head at each *y* position is given by Eq. 3-4-6. In Sect. 3.5, we consider as if the step motion of the catalytic domain occurs at the definite position named *y*_c_. In this section, we discuss the muscle stiffness on the same simplified scheme. Since *y* changes with time at the constant velocity, the stress *p* produced by one head per one step is given by integration by *y*. Then, referring to Figure 7(a) or (b),

(4-1-1) $$p=(1/L)[\int_{y_{c-L}}^{0}(-\kappa_{\text{f}}y)\text{d}y+\int_{0}^{y_{c}}(-\kappa_{\text{b}}y)\text{d}y]\text{.}$$

Thus we obtain

(4-1-2) $$p=(1/2L)\{\kappa_{\text{f}}L^{2}\;-2\;\kappa_{\text{f}}Ly_{\text{c}}+(\kappa_{\text{f}}-\kappa_{\text{b}})y_{\text{c}}\}\text{.}$$

From Eq.4-1-2, *y*_c_ is given by

(4-1-3) $$y_{c}=L[\kappa_{\text{f}}-\;\{\kappa_{\text{f}}^{2}-(\kappa_{\text{f}}-\kappa_{\text{b}})(\kappa_{\text{f}}-\;(2p/L)){\}}^{1/2}]/\text{ (}\kappa_{\text{f}}-\kappa_{\text{b}})$$

Let us denote *y*_c_ at *p* = *p*_0_ as *y*_c0_ and *y*_c_ at *p* = 0 as *y*_c_(0), then from Eq. 4-1-3,

(4-1-4) $$y_{\text{c}\;0}=L[\kappa_{\text{f}}\;-\{\kappa_{\text{f}}^{2}-(\kappa_{\text{f}}-\kappa_{\text{b}})\text{ (}\kappa_{\phi }-\;(2p_{0}/L)){\}}^{1/2}]/\text{ (}\kappa_{\text{f}}-\kappa_{\text{b}}),$$

(4-1-5) $$y_{\text{c}}(0)=L\text{ \{}\kappa_{\text{f}}-\;(\kappa_{\text{f}}\kappa_{\text{b}}{)}^{1/2}\}/(\kappa_{\text{f}}-\kappa_{\text{b}})\text{.}$$

The stiffness is κ_f_ in the region *y*_c_ − *L* < *y* < 0 and κ_b_ in the region 0 < *y* < *y*_c_, and *y* changes with time at the constant velocity. Hence, denoting the average stiffness as *s*, we have

(4-1-6) $$s=\text{ \{}\kappa_{\text{f}}(L\text{ -}y_{\text{c}})+\kappa_{\text{b}}\;y_{\text{c}}\}/L\text{.}$$

Eqs. 4-1-3 and 4-1-6 give the stiffness *s* as function of *p*. Based upon the assumption that the proportion *r* of the number of myosin heads that are simultaneously attached to an actin is a constant independent of *P* (Eq. 3-1-1), we have *p*/*p*_0_ = *P*/*P*_0_ (3-1-2). By the same reason we have

(4-1-7) $$s/s(p_{0})=S/S(P_{0}),$$

where *S* is the muscle stiffness, and *s*(*p*_0_) and *S*(*P*_0_) are, respectively, *s* and *S* at *P* = *P*_0_. Then, from Eqs. 4-1-6 and 4-1-7, we have

(4-1-8) $$S/S(P_{0})=\;\{\kappa_{\text{f}}(L\text{ -}y_{\text{c}})+\kappa_{\text{b}}y_{\text{c}}\}/\{\kappa_{\text{f}}(L\text{ -}y_{\text{c}0})+\kappa_{\text{b}}y_{\text{c0}}\}\text{.}$$

**Figure 9.**
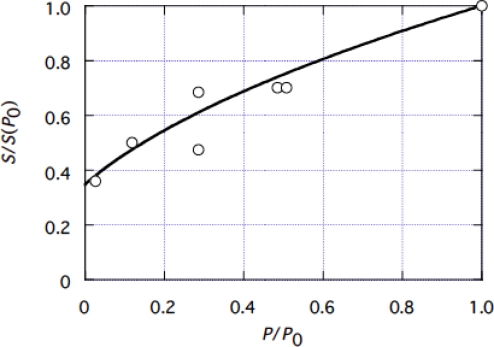
Relative stiffness *S*/*S*(*P*_0_) of muscle as a function of *P*/*P*_0_. Open circles: Experimental data cited from Figure 10B of a paper by Ford *et al*. [10]. Solid line: calculated by Eq. 4-1-8.

The ratio *S*/*S*(*P*_0_) was experimentally determined by Ford *et al*. [10], as shown by circles in Figure 9.

The muscle stiffness *S* for *P* = 0 is denoted as *S*(0). The ratio *S*(0*)*/*S*(*P*_0_) is 0.35 as seen in Figure 9:

(4-1-9) $$S(0)/S(P_{0})=0.35.$$

Then, from Eq. 4-1-8,

(4-1-10) $$\{\kappa_{\text{f}}(L\text{ -}y_{\text{c}}(0))+\;\kappa_{\text{b}}y_{\text{c}}(0)\}/\{\kappa_{\text{f}}(L\text{ -}y_{\text{c}0})+\kappa_{\text{b}}\;y_{\text{c}0}\}=0.35.$$

Since *L* = 5.46 nm (Appendix) and *p*_0_ = 5.7 pN (Eq. 2-2-3), there are four unknown parameters (κ_f_, κ_b_, *y*_c0_, *y*_c_(0)) which are related by the three equations 4-1-4, 4-1-5 and 4-1-10. Hence, if one of the four is determined the other three can be determined. Since *y*_c_(0) is *y*_c_ for the fastest contraction, Figure 7(a) suggests that *y*_c_(0) is relatively close to *L* (5.46 nm). Calculations are made for κ_f_, κ_b,_*y* _c0_, and *y*_c_ by Eq. 4-1-3, and *S*/*S*(*P*_0_) by Eq. 4-1-8 with various trial values of *y*_c_(0). After several calculations, good agreement with experimental data was obtained with

(4-1-11) $$y_{\text{c}}(0)=4.2\text{ nm}\text{.}$$

Calculated result is shown by the curve in Figure 9. Agreement with the experimental data is fairly good. The other parameters are determined using the value of *y*_c_(0) in Eq. 4-1-11 as

(4-1-12) $$y_{\text{c}0}=0.73\text{ nm,}$$

(4-1-13) $$\kappa_{\text{f}}=2.80\text{ pN/nm},$$

(4-1-14) $$\kappa_{\text{b}}=0.26\text{ pN/nm}\text{.}$$

**Figure 10.**
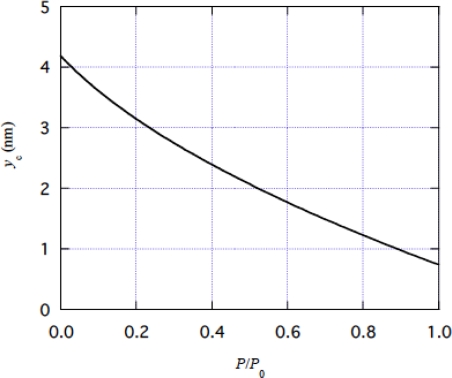
*y*_c_ as a function of *P*/*P*_0_, calculate by Eq. 4-1-3.

Now we can calculate *y*_c_ as a function of *P*/*P*_0_ by Eq.4-1-3. Figure 10 shows calculation result. This *y*_c_ vs. *P*/*P*_0_ relation will be used for various calculations.

### 4.2 Force-velocity relation

The probability *Q* that the myosin head moves over the potential barrier U* is expressed by *Q* = *A*exp(−U*/*kT*) (Eq. 3-5-1) where U* = U*_0_ − *by* (Eq. 3-5-4) for *y* > 0. Although some complexity is expected around the isometric tension *P*_0_ as discussed concerning Figure 6(c), these expressions are assumed to hold for *y* > *y*_c0_, where *y*_co_ becomes *y*_c_ at *P*_0_. It follows from the above equations that *Q* is proportional to exp(*by*/*kT*), and most transitions for the head to cross over U* are expected to occur around *y*_c_ as discussed in Sect. 3. 5. Then the mean time *t*_c_ needed to complete the transition is approximately given by the inverse of *Q*:

(4-2-1) $$t_{\text{c}}=(1/A)\exp(U*(y_{\text{c}})/kT),$$

where

(4-2-2) $$U*(y_{\text{c}})=U{*}_{0}-\;by_{\text{c}}\text{.}$$

From the other viewpoint, the transition is approximately completed while *y* changes from *y*_c0_ to *y*_c_ with the velocity *v*, and thus *t*_c_ can be expressed by

(4-2-3) $$t_{\text{c}}=(y_{\text{c}}\;-\;y_{\text{c0}})/v\text{.}$$

From Eqs. 4-2-1, 4-2-2 and 4-2-3, the velocity *v* is given by

(4-2-4) $$v=B(y_{\text{c}}\;-\;y_{\text{c}0})\exp(by_{c}/kT),$$

where

(4-2-5) $$B=A\exp\left(-{U*}_{0}/kT\right)\text{.}$$

The velocity *v* can be calculated as a function of *P*/*P*_0_ when *B* and β are given, as the values of *y*_c_ are given in Figure 10.

**Figure 11.**
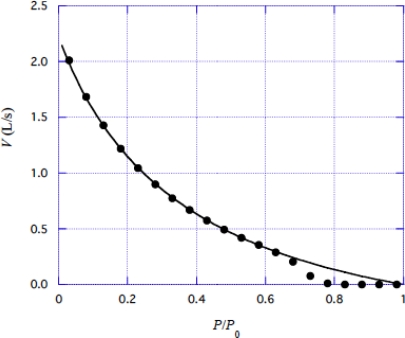
Force-velocity relation. Black circle are experimental data by Edman [31]. Solid line shows calculation result by Eq. 4-2-4.

Edman [31] studied the force-velocity relation of frog muscle fibers in detail. The experimental results were represented by empirical equation (2) in [31]. Values calculated by the empirical equation are shown by black circles in Figure 11. Calculations based upon Eq.4-2-4 were done with various trial values of *B* and β to get the best fit with the experimental data. The curve in Figure 11 shows the results by using the parameter values:

(4-2-6) $$B=1.92\times 10^{8}{\;}^{\text{-1}},$$

(4-2-7) $$b/kT=0.29{\text{ (nm)}}^{\text{-1}}\text{.}$$

Agreement with the experimental data is good for *P*/*P*_0_ < 0.7, but there is a distinct deviation from the calculation curve in larger *P*/*P*_0_ region. In Sect. 3.6, the pull-up mechanism was considered as a possible origin of the deviation.

### 4.3 Energy liberation rate

Energy liberation rate in the crossbridge mechanism is denoted as *W* + *H* per unit volume of muscle per unit time. Homsher *et al*. [40] determined the energy production in contracting muscles from myothermal and mechanical measurements at *V* = *V*_max_, *V*_max_/2, and 0. It is assumed that 30% of the heat production at *P* = *P*_0_, which is 4.4 kW/m^3^, comes from a non-crossbridge source and this amount is subtracted from the experimental values, with the results

(4-3-1a) $$W+H=35.2{\text{ kW/m}}^{3}\text{at}\;P/P_{0}=0,$$

(4-3-1b) $$=51.8{\text{ kW/m}}^{3}\text{ at}\;P/P_{0}=0.21,$$

(4-3-1c) $$=10.2{\text{ kW/m}}^{3}\text{ at}\;P/P_{0}=1.00.$$

**Figure 12.**
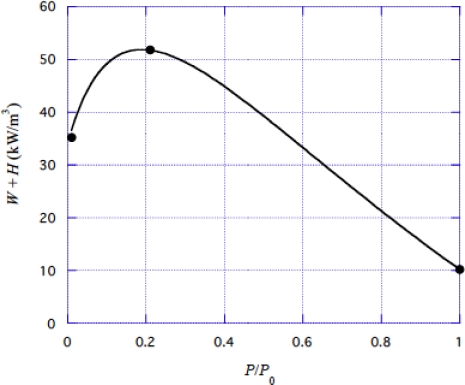
Energy liberation rate *W* + *H* in contracting muscle as a function of *P*/*P*_0_. Filled circles: experimental data by Homsher *et al*. [40] (cf. Eq. 4-3-1). Curve: calculated by Eq. 4-3-13.

These values are shown by filled circles in Figure 12.

*W* + *H* consists of two components: one is directly related with the force production and denoted as (*W* + *H*)_1_. Generally there is an energy loss in working machine, which is called the maintenance heat for muscle and denoted as *H*_0_:

(4-3-2) $$W+H={\left(W+H\right)}_{1}+H_{0}\text{.}$$

(*W* + *H*)_1_ consists of two parts. A fraction of ε_ATP_ is spent for the force production by each head attached to actin during crossing over U*. Let us denote this fraction of *W* + *H* as (*W* + *H*)_1,1_. For such force production, the complexes (MA_3_) have to be formed. A fraction ofε_ATP_ is used firstly to create (MA_3_) in the process from Figure 3(a) to (b) and lastly to decompose the complex. Let us denote the fraction of *W* + *H* used for these processes as (*W* + *H*)_1,2_. Then

(4-3-3) $${\left(W+H\right)}_{1}={\left(W+H\right)}_{1,1}\text{ +}{\left(W+H\right)}_{1,2}\text{.}$$

Let us also denote the portion of ε_ATP_ that the head spends to produce force during each step motion as *g*. Then (*W* + *H*)_1,1_ is given by

(4-3-4) $${\left(W+H\right)}_{1,1}=(v/L)(rN)g,$$

where (*v*/*L*) is the number of the force-production cycle of each head per unit time, *N* is the number of myosin heads in a unit volume of muscle, and hence *rN* is the number of myosin heads in a unit volume which are simultaneously attached to actin filaments. Since the head has to cross over the potential barrier U* to produce force, *g* will increase with increasing U*. As an approximation, *g* is set as

(4-3-5) $$g=B_{g}\;U*(y_{\text{c}}),$$

where *B_g_* is a constant. Thus, by Eq.3-5-4,

(4-3-6) $$g=B_{\text{g}}(U{*}_{0}-by_{\text{c}}),$$

where *b* is given by Eq. 4-2-7. From Eqs. 4-3-4 and 4-3-6

(4-3-7) $${\left(W+H\right)}_{1,1}=(B_{g}rN/L)(U{*}_{0}-by_{\text{c}})v\text{.}$$

As for (*W* + *H*)_1,2_, the frequency of the attachment-detachment of all the heads in unit volume is given by (*W* + *H*)_1_/ε_ATP_. Let the energy spent by each head for the attachment-detachment be *h*_1_, then

(4-3-8) $${\left(W+H\right)}_{1,2}=h_{1}{\left(W+H\right)}_{1}/{ɛ}_{\text{ATP}}\text{.}$$

Thus Eq. 4-3-3 becomes

(4-3-9) $${\left(W+H\right)}_{1}=h_{1}{\left(W+H\right)}_{1}/{ɛ}_{\text{ATP}}+{\left(W+H\right)}_{1,1}\text{.}$$

That is,

(4-3-10) $${\left(W+H\right)}_{1}={\left(W+H\right)}_{1,1}/(1\text{ -}h/{ɛ}_{\text{ATP}})\text{.}$$

Thus Eq. 4-3-2 becomes

(4-3-11) $$W+H=(C_{g}rN/L)(U{*}_{0}-by_{\text{c}})v+H_{0},$$

where

(4-3-12) $$C_{g\cdot }=B_{\text{g}}/(1\text{ -}h/{ɛ}_{\text{ATP}})\text{.}$$

The maintenance heat *H*_0_ is assumed to be a constant as was done by Hill [41], and set equal to *W* + *H* at *v* = 0, *i. e*., 10.2 kW/m^3^ given in Eq. 4-3-1c. Then, Eq. 4-3-11 becomes

(4-3-13) $$W+H=(C_{g}rN/L)(U{*}_{0}-by_{\text{c}})v+10.2\times 10^{3}{\text{ (W/m}}^{3})\text{.}$$

*W* + *H* was calculated for various values of *C_g_* and U*_0_ with the value of *b* in Eq. 4-2-7. The best agreement was obtained with the parameter values:

(4-3-14) $$C_{g}=1.69,$$

(4-3-15) $$U{*}_{0}/kT=1.34.$$

Calculated values of *W* + *H* are shown by the curve in Figure 12. Agreement with the experimental data is good.

**Figure 13.**
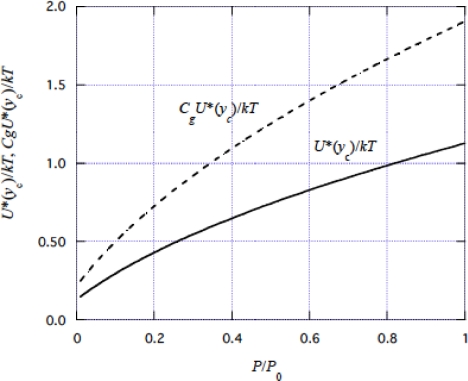
Calculated U*(*y*_c_)/*kT* and *C*_*g*_U*(*y*_c_)/*kT* as functions of *P*/*P*_0_. U*(*y*_c_) = U*_0_ – *by*_c_ (Eq. 4-2-2) and *C*_*g*_ is 1.69 (Eq 4-3-14). The myosin head crosses over the potential barrier U*(*y*_c_) (cf, Figure 2(d)) and produces force. Energy *g* spent at each step of this force generation is expected to be between U*(*y*_c_) and *C*_*g*_U*(*y*_c_).

Now we can calculate U*(*y*_c_) = U*_0_ − *by*_c_ (Eq. 4-2-2) by using *y*_c_ in Figure 10, and U*_0_ in Eq. 4-3-15 and *b* in Eq. 4-2-7. Calculation result is shown by the solid curve in Figure 13.

Hill [41] gave the empirical expression of *W* +*H* by *W* + *H* = (0.16*P*_0_ + 1.18*P*)*v* + *H*_0_. It should be noted that Eq. 4-3-13 has the same functional form as Hill's expression when (U*_0_−*by*_c_) in Figure 13 is approximated by a linear function of *P*.

The portion of ε_ATP_ used for each force production step, *g*, is expressed by B_g_U*(*y*_c_) (Eq. 4-3-5). Since *C_g_*. =*B_g_*/(1 − *h*/ε_ATP_) (Eq.4-3-12), *B_g_* < *C_g_* and thus *g* < C_g_U*(*y*_c_). With *C_g_* = 1.69 (Eq. 4-3-14), C_g_U*(*y*_c_) was calculated and the result is shown by dashed curve in Figure 13. Values of *g* are expected to be between the solid and dashed curves.

From Eq. 2-1-4, the traveling distance *D* of a myosin head in muscle is given by

(4-3-16) $$D=r{ɛ}_{\text{ATP}}N_{\text{hs}}V/(W+H)\text{.}$$

**Figure 14.**
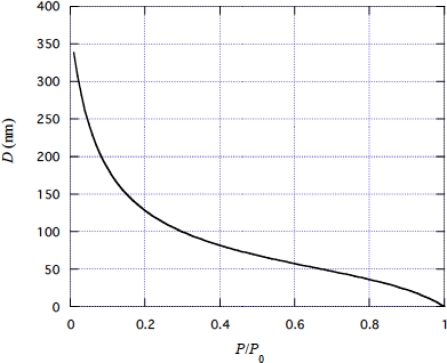
*D* vs. *P*/*P*_0_ relationship at about 0°C calculated using Eq. 4-3-16. *D* is the distance over which a myosin head translates using ε_ATP_.

*D* is calculated by using the values of *V* in Figure 11 and *W* + *H* in Figure 12. Results are shown in Figure 14. The myosin traveling distance *D* is 369 nm in the free shortening at 0°C, which means that a myosin head repeats 68 steps during one ATP hydrolysis cycle as *D*/*L* = 369/5.46 = 68.

### 4. 4 Isometric tension transient

Huxley and Simmons [3] and Ford *et al*. [33] studied the response of frog muscle fiber to stepwise length changes. They divided the tension response to quick length changes into four phases. Phase 1 is the initial response, *i.e*. a sudden step-change in tension. After this step is completed, a rapid partial recovery toward the original tension (phase 2, the early recovery phase) occurs, followed by a slowdown of recovery (phase 3), and finally a much slower return to the original tension (phase 4). Among them phases 1 and 2 were studied mathematically based on a new model in [6] and [7]. In this section their studies are outlined.

The fastest response, phase 1, seems to be related with elastic length changes in the crossbridge. The muscle length is controlled from outside in the isometric tension transient and the initial and final states are the stationary state. It is assumed that, in the intermediary, the barrier U* between wells 2 and 3 in Figure 2(d) is kept as high as in the stationary state. Then phase 2 corresponds to the process of adjustment of the head population between wells 2 and 3. The slower processes, phases 3 and 4, seem to be related with the U* transition caused by thermal fluctuation. (The pull-up effect in Figure 7(c) is neglected here.)

To discuss phase 2, potential distribution other than the vicinity of wells 1 and 2 can be treated as infinity since there is practically no translation over U*. Since the potential distribution in Figure 2(d) is for a single myosin head, the potential distribution corresponds to chemical affinity between a myosin head and actin molecules. This potential, which is named affinity potential, is represented by *U*_af_. Now we consider heads in a muscle, where the head is under the influence of the elastic force *f*_J_. The nature of *f*_J_ is already known through Eq. 3-4-5. The effect of *f*_J_ is discussed by using an elastic potential *U*_el_. Thus the total potential energy *U* of the head is given by

(4-4-1) $$U=U_{\text{af}}+U_{\text{el}}\text{.}$$

**Figure 15.**
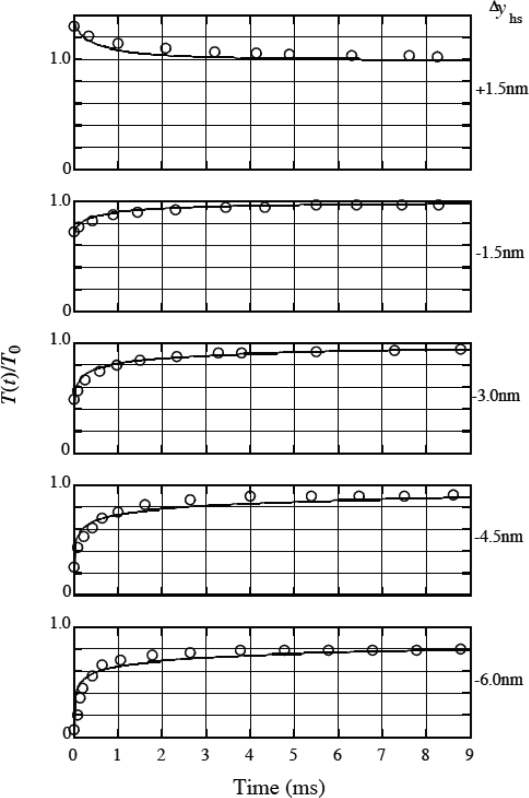
*T*(*t*)/*T*_0_ as a function of time *t* for various length change steps Δ*y*_hs_ in the isometric tension transient. *T*(*t*): tension *P* in phase 2. *T*_0_: the isometric tension *P*_0_. Circles: experimental data cited from Figure 23 in the article by Ford *et al*. [33]. Solid lines: Calculation results reported in [7].

The stepwise length changes cause a step-change in tension and thus a step-change in *U*_el_. Then the ratio of head populations in wells 1 and 2 will alter so that *U*_el_ changes toward the value at the stationary isometric state. The changes in population and tension are calculated as functions of time based upon the statistical mechanics in [6] and [7]. Calculated tension recovery in phase 2 are shown by the curves in Figure 15 for various quick length changes per half sarcomere denoted as Δ*y*_hs_. Here, the notations used by Ford *et al*. [30] are used: *T*(*t*) represents tension as a function of time *t* and *T*_0_ is the isometric tension. *T* and *T*_0_ are the same as *P* and *P*_0_, respectively, in the preceding sections. The experimental data on *T*(*t*)/*T*_0_ by Ford *et al*. [33] are shown by circles in Figure 15. The curves represent a general tendency of the experimental data.

**Figure 16.**
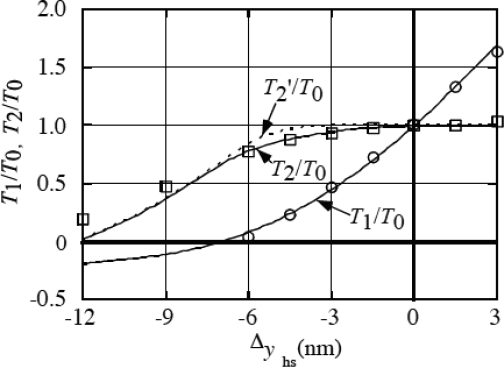
*T*_1_/*T*_0_ and *T*_2_/*T*_0_ as functions of Δ*y*_hs_. Circles and squares: experimental data cited from Figure 13 of Ford *et al*. [33]. Solid and dotted lines are calculation results reported in [7]. *T*_0_ is the isometric tension *P*_0_, *T*_1_ is for *P* just after the quick length change, *T*_2_ is for *P* at *t* = 9 ms (the maximum time in Figure 15) and *T*_2_’ is for *P* at *t* = ∞.

Values of *T*_1_/*T*_0_, *T*_2_/*T*_0_ and *T*_2_/*T*_0_ were obtained for various length changes Δ*y*_hs_ [7], where *T*_1_ is for *P* just after the quick length change, *T*_2_ is for *P* at *t* = 9 ms (the maximum time on the abscissa in Figure 15) and *T*_2_’ is for *P* at *t* = ∞. Results are shown for *T*_1_/*T*_0_ and *T*_2_/*T*_0_ by solid curves and *T*_2_’/*T*_0_ by dotted curve in  Figure 16. The experimental data by Ford *et al*. [33] are shown by circles for *T*_1_/*T*_0_ and by squares for *T*_2_/*T*_0_. The solid curves well reproduce the experimental data. The calculation results are similar to those reported by Ford et al [33], although the model is different.

### 4. 5 Isotonic Velocity Transient

**Figure 17.**
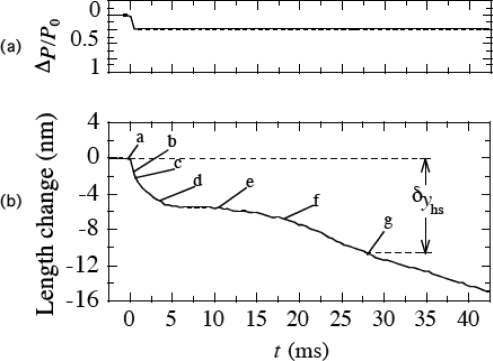
Illustration for the isotonic velocity transient, drawn following Figure 7 of Huxley [36]. The abscissa is time in ms. (a) Load change is shown by the relative load Δ*P*/*P*_0_ = (*P*_0_−*P*)/*P*_0_, which is suddenly altered at *t* = 0 and then kept constant. (b) Time course of length change.

As mentioned in Sect.3.10, isotonic velocity transients were studied by Podolsky [34], Civan and Podolsky [35] and Huxley [36]. Muscles were stimulated and initially held at a constant length. It was then released suddenly and allowed to shorten under a constant load. The transient response is illustrated in Figure 17 referring to Figure 7 of Huxley's article [36]. The sudden load change Δ*P* is applied at *t* = 0 and then *P* is kept constant as shown in Figure 17 (a). Length change is shown in Figure 17(b). As the first response, a sudden shortening a→b occurs as shown in Figure 17(b). It is followed by a shortening b→c→d with a speed several times higher than the steady-state speed appropriate for the load. The speed then declines to a low value (d→e) and subsequently tends to increase (e→f) and reach its steady-state value around g. A characteristic feature of the curve is the downward convex around d. As mentioned by Civan and Podolsky [35], it seems difficult to explain this characteristic with the Huxley model.

Discussion on the isotonic velocity transient in Sect.V of [7] is somewhat complex, and only its summary is given here. The first response of muscle to the sudden change of load is a release of tension of crossbridges, which cause the rapid filament sliding, corresponding to a → b in Figure 17(b). In the new model, this filament sliding will cause an increase in *y*, and its upper limit *y*_c_ increases from *y*_c0_ to a new value *y*_c_’. Then the heads in *y*_c0_ < *y* < *y*_c_’ will cross over U* with relevant time constants, causing a filament sliding. This process seems to correspond to b→ c → d in Figure 17(b). The filament sliding changes the elastic potential *U*_el_ (cf. Eq. 4-4-1) in wells 1 and 2 in Figure 2(d). As in the case of isometric transient, this change of *U*_el_ will change the ratio of head populations in wells 1 and 2, producing a balance between the outside and inside stresses. The balance is realized around e in Figure 17(b) and the filament sliding stops. During these processes, however, a few heads will cross over U* by thermal fluctuation and cause slight filament sliding which decreases the number of heads in wells 1 and 2. There is a lower limit of the sum of the numbers of heads in wells 1 and 2 to keep the balance of stresses. This limit comes around e, and the filament sliding become evident around f. The sliding induces positive *f*_J_ and reduces U*, causing further increase of the sliding velocity. The sliding velocity gradually increases by such feedback and finally reaches the steady value around g.

## 5. Additional comments

### 5.1 On the large values of D/r

Some readers of [7] commented that the *D*/*r* value of 900 nm in Eq. 2-1-5 seems too large to believe. This section is prepared to mention that large *D*/*r* values can be obtained also from other experimental data.

Yanagida *et al*.[42] studied crab muscle from which Z membranes are deleted. Based upon the obtained data, they proposed that *D* is as large as more than 60 nm in the unloaded condition. Harada *et al*.[43] observed movement of very short actin filaments on a glass strip covered by myosin filaments, and proposed that *D* > 60 nm at 30 °*C* and *D* >200 nm at 20°C. As mentioned by several authors, however, it is difficult to obtain *D* values from their experimental data without speculation. It is, however, possible to obtain *D*/*r* from their experimental data [44]. A basic idea to derive *D*/*r* in [44] is similar to that in deriving Eq. 2-1-4, and only essentials and final results of [44] are presented below. The *D*/*r* value of 900 nm was obtained from the relation *D*/*r* =ε_ATP_ *N*_hs_*V* /(*W* + *H*) (Eq. 2-1-4) using the macroscopic values of *N*_hs_, *V* and (*W* + *H*). We can find relevant microscopic quantities in the experimental results by Yanagida *et al*. [42] and Harada *et al*.[43].

Giving their notations in parenthesis, Yanagida *et al*. [42] determined the filament sliding velocity (*V*_F_), ATP activity of sarcomere (*V^s^*_ATP_) and the average number of myosin molecules in half a thick filamenit overlapping with thin filament during sliding (*N*_m_). *D*/*r* values are calculated by using these experimental data. Obtained *D*/*r* are 10600 nm at 5°C and 9800 nm at 15°C. These *D*/*r* values are very large presumably because the removal of Z membranes causes a large inter-filament distance and thus small *r*.

Harada *et al*. [43] determined the filament sliding velocity (*V*_F_), the number of ATP molecules spent per unit length of an actin filament per unit time (d*P*_i_/d*t*). There is some uncertainty in determining the number of myosin heads which can bind to actin molecules per 1 m of actin filament and is set equal to1.7×10^8^ as the most probable number in calculation [44]. The obtained values of *D*/*r* are 1280 nm at 22°C and 380 nm at 30°C. These *D*/*r* values are of the same order of magnitude as 900 nm.

### 5.2 On the two-headed structure of myosin molecule

It is assumed that 41% of myosin heads are attached to actin in contracting muscles (Eq. 3-1-1). Let us check whether or not this percentage is reasonable in connection with the actual muscle structure.

There are two actin filaments per myosin filament in frog skeletal muscle. An actin filament has two actin molecules per 5.46 nm. A myosin filament has three myosin molecules per 14.3 nm. Consequently, the relevant densities per nm are 2×2/5.46 = 0.73/nm for actin molecule and 3×2/14.3 = 0.42/nm for myosin head. If 41% of the heads are attached to actin, the density of the attached myosin head is 0.42×0.41 = 0.17/nm. Therefore, available actin molecules per attached myosin head is 0.73/0.17 = 4.3. If the head uses 3 out of the 4.3 actin molecules to form the complex MA_3_, the remaining 1.3 actin molecules will provide a space which makes movement of MA_3_ possible. Hence, the figure 4.3 means almost the full use of actin molecules for the filament sliding. As already mentioned in Sect. 2. 2, by X-ray diffraction study, Matsubara *et al*. [12] estimated that about 80% of myosin heads migrate around the thin filament in isometrically contracting muscle. Higher values for this percentage (about 92~99%) is reported by Yagi *et al*. [13]. Since these percentages are about twice *r* = 41%, it can be speculated that one of two heads of a myosin molecule attaches to actin and produces force while the other exists in the vicinity of the thin filament as non-attached relief. The 4.3 actin molecules available for one attached head will be used efficiently if the relief head exists nearby and uses them when the attached head is energetically exhausted. The two-headed structure of a myosin molecule seems to be a device to produce force efficiently.

### 5.3 On cytoplasmic streaming in Characean algae

Concerning the cooperativity of myosin heads discussed in Sect.3.7, the observation by Nothnagel and Webb [45] is interesting. They observed that dispersed myosin cannot drive cytoplasmic streaming in Characean algae while myosin on an endoplasmic network can easily drive streaming. The cooperativity of myosin heads in shortening muscle is made possible as they bind to the same myosin filament. Similarly, endoplasmic network will be necessary for myosin molecules to cooperate to produce cytoplasmic streaming in Characean algae.

Myosin heads move as fast as 60μm/s in the algae, *Nitella* [46] and *Chara* [47], compared to about 2.4μm/s of filament sliding velocity under no load in muscle (cf. Appendix). Since the velocity *v* is proportional to exp(−U*(*y*_c_)/*kT*) by Eqs. 4-2-4, 4-2-2, and 4-2-5 in the new model, relatively small change in U* causes very large change in *v*. It seems possible that various actomyosin systems have developed for various purposes by changing U*. Presumably U* is low in *Nitella* and *Chara* where velocity is important, while U* is relatively high in muscle where force is important.

## 6. Summary

The new model described in the present paper is characterized by the constant *r* (Eq.3-1-1), the formation of the polaron-like compound of MA_3_ (Sect. 3. 3), the nonlinear elastic property of the crossbridge (Eq. 3-4-6), and the *f*_J_-dependence of of U* (Eq.3-5-2) which reflects cooperativity among myosin heads in shortening muscle (Sect. 3.7).

Calculations based on the model well explain experimental data on muscle stiffness (Figure 9), force-velocity relation (Figure 11), energy liberation rate (Figure 12) and the time courses in transient phenomena (Figures 15~ 17). Although various parameter adjustments are made in these calculations, it should be noted that agreement between calculation results and experimental data is possible only when the characteristic features of the functional form derived by the model are similar to those of the experimental data.

In the power stroke model, an actin filament is treated as a relatively passive element like a ladder for a myosin head. In general, however, protein molecules have their proper active functions in biological systems. There was a question of why an actin molecule plays such relatively passive role in muscle. In the new model, actin molecules play more active roles in mutual cooperation with myosin.

The structure of a protein molecule is polar, i. e., the center of symmetry is lacking in its structure. Generally mechanical properties of the polar systems are treated as four-variable systems i. e., with strain, stress, electric field and polarization. Two of the four (e. g., stress and electric field) are adopted as independent variables and the others are dependent variables (cf, e.g., [8]). Recently the present authors [48] have shown that the properties of the flagellar motor can be well explained by treating them as four-variable system. As symbolically shown by + and − in Figure 2(c) and (d), it seems plausible that electric charges and polarization implicitly play important roles in muscles.

## Appendix

### Fundamental Parameter Values

*kT*: 3.77×10^−21^ J at 0°C
*L*: period of actin strand projection onto the filament axis: 5.46 nm
*N*: number of myosin heads in 1 m ^3^, calculated with *N*_hs_ and *s*: :1.68×10^23^ m^−3^
*N*_hs_: number of myosin heads in a volume with a base of 1 m^2^ and a thickness of half the sarcomere length : 1.76×10^17^ m^−2^ [49]
*P*_0_: isometric tension : 4.1×10^5^ N/m^2^ [31]
*p*_0_: mean force produced by one myosin head in isometric contraction: 5.7×10^−12^ N/head [11], (Eq. 2-2-3)
*r*: ratio of the number of myosin heads simultaneously in the attached state to the number of all the heads: 0.41 (Eq. 3-1-1)
*s*: sarcomere length: 2.10 μm [31]
*V*_max_: shortening velocity under no load near 1.8 °C: 2.25(*L*/*s*) [31]
*v*_max_: velocity of filament sliding under no load in muscle at 1.8°C: 2.36 μm/s [31]
*y*_c_(0): critical *y* at free shortening : 4.2 nm (Eq. 4-1-11)
*y*_c0_: critical *y* at the isometric tension : 0.73 nm (Eq. 4-1-12)
ε_ATP_: energy liberated by the hydrolysis of one ATP molecule: 8.0×10^−20^ J/molecule [50], 21*kT* at 0°C
κ_f_: stiffness of crossbridge when the myosin head exertes positive force on a myosin filament: 2.80×10^−3^ N/m = 2.80 pN/nm (Eq. 4-1-13)
κ_b_: stiffness of crossbridge when the myosin head exerts negative force on a myosin filament: 0.26×10^−3^ N/m = 0.26 pN/nm (Eq. 4-1-14)

## References

[1] Huxley AF (1957). Muscle structure and theories of contraction. Progr. Biophys. Biophys. Chem..

[2] Huxley HE (1969). The mechanism of muscular contraction. Science.

[3] Huxley AF, Simmons RM (1971). Proposed mechanism of force generation in striated muscle. Nature.

[4] Huxley AF (2000). Mechanics and models of the myosin motor. Phil. Trans. R. Soc. Lond. B.

[5] Mitsui T, Chiba H (1996). Proposed modification of the Huxley-Simmons model for myosin head motion along an actin filament. J. Theor. Biol..

[6] Mitsui T, Kumagai S, Chiba H, Yoshimura H, Ohshima H (1998). Induced potential model for musculat contraction mechanism, including two attached states of myosin head. J. Theor. Biol..

[7] Mitsui T (1999). Induced potential model of muscular contraction mechanism and myosin molecular structure. Adv. Biophys..

[8] Mitsui T, Tatsuzaki I, Nakamura E (1976). An introduction to the physics of ferroelectrics.

[9] Geeves MA, Holmes KC (2005). The molecular mechanism of muscular contraction. Advance in Protein Chemistry.

[10] Ford LE, Huxley AF, Simmons RM (1985). Tension transients during steady shortening of frog muscle fibres. J. Physiol..

[11] Ishijima A, Harada Y, Kojima H, Funatsu T, Higuchi H, Yanagida T (1994). Single-molecule analysis of the actomyosin motor using nano-manipulation. Biochem. Biophys. Res. Comm..

[12] Matsubara I, Yagi N, Hashizume H (1975). Use of an X-ray television for diffraction of the frog striated muscle. Nature.

[13] Yagi N, Takemori S, Watanabe M (1993). An X-ray diffraction study of frog skeletal muscle during shortening near the maximum velocity. J. Mol. Biol..

[14] Podolsky RJ, Onge SSt, Yu L, Lymn RW (1976). X-ray diffraction of actively shortening muscle. Proc. Natl. Acad. Sci. USA.

[15] Huxley HE, Sugi H, Pollack GH (1979). Time resolved X-ray diffraction studies in muscle. Crossbridge Mechanism in Muscle Contraction.

[16] Huxley HE, Kress M (1985). Crossbridge behaviour during muscle contraction. J. Musc. Res. Cell Motility.

[17] Molloy JE, Burns JE, Kendrick-Jones J, Tregear RT, White DCS (1995). Movement and force produced by a single myosin head. Nature.

[18] Kitamura K, Tokunaga M, Hikikoshi-Iwane A, Yanagida T (1999). A single myosin head moves along an actin filament with regular steps of 5.3 nanometres. Nature.

[19] Ramsey RW, Street SF (1940). The isometric length-tension diagram of isolated skeletal muscle fibers of the frog. J. Cell. Comp. Physiol..

[20] Andreeva AL, Andreev OA, Borejdo J (1993). Structure of the 265-kilodalton complex formed upon EDC cross-linking of subfragment 1 to F-actin. Biochem..

[21] Xiao M, Andreev OA, Borejdo J (1995). Rigor crossbridges bind to two actin monomers in thin filaments of rabbit psoas muscle. J. Mol. Biol..

[22] Kittel C (1986). Introduction to Solid State Physics.

[23] Rayment I, Rypniewski WR, Schmidt-Bäse K, Smith R, Tomchick DR, Benning MM, Winkelmann DA, Wesenberg G, Holden HH (1993). Three-dimensional structure o myosin subfragment-1: a molecular motor. Science.

[24] Rayment I, Holden HM, Whittaker M, Yohn CB, Lorenz M, Holmes KC, Milligan RA (1993). Structure of the actin-myosin complex and its implications for muscle contraction. Science.

[25] Burgess SA, Walker ML, White HD, Trinick J (1997). Extensibility within myosin heads revealed by negative strain and single-particle analysis. J. Cell Biol..

[26] Wakabayashi K, Yagi N (1999). Muscle contraction: challenges for synchrotron radiation. J. Synchrotron Rad..

[27] Mandelson RA, Morales MF, Botts J (1973). Segmental flexibility of the S-1 moiety of myosin. Biochem..

[28] Elliott A, Offer G (1978). Shape and flexibility of the myosin molecule. J. Mol.Biol..

[29] Walker M, Knight P, Trinick J (1985). Negative staining of myosin molecules. J. Mol. Biol..

[30] Eyring H (1936). Viscosity, plasticity, and diffusion as examples of absolute reaction rate. J. Chem. Phys..

[31] Edman KAP (1988). Double-hyperbolic force-velocity relation in frog muscle fibres. J. Physiol..

[32] Lymn RW, Taylor EW (1971). Mechanism of adenosin triphosphate hydrolysis by actomyosin. Biochemistry.

[33] Ford LE, Huxley AF, Simmons RM (1977). Tension responses to sudden length change in stimulated frog muscle fibres near slack length. J. Physiol..

[34] Podolsky RJ (1960). Kinetics of molecular contraction: the approach to steady state. Nature.

[35] Civan MM, Podolsky RJ (1966). Contraction kinetics of striated muscle fibres following quick changes in load. J. Physiol..

[36] Huxley AF (1974). Muscular contraction. J. Physiol..

[37] Huxley HE, Steward A, Sosa H, Irving T (1994). X-ray diffraction measurements of the extensibility of actin and myosin filaments in contracting muscle. Biophys. J..

[38] Wakabayashi K, Sugimoto Y, Tanaka H, Ueno Y, Takazawa Y, Amemiya Y (1994). X-ray diffraction evidence for the extensibility of actin and myosin filaments during muscle contraction. Biophys. J..

[39] Irving M (1995). Give in the filament. Nature.

[40] Homsher E, Irving M, Yamada T, Pollack GH, Sugi H (1984). The effect of shortening on energy liberation and high energy phosphate hydrolysis in frog skeletal muscle. Contractile Mechanism in Muscle.

[41] Hill AV (1964). The effect of load on the heat of shortening muscle. Proc. Roy. Soc. B.

[42] Yanagida T, Arata T, Oosawa F (1985). Sliding distance of actin filamentinduced by a myosin crossbridge during one ATP hydrolysis cycle. Nature.

[43] Harada Y, Sakurada K, Aoki T, Thomas DD, Yanagida T (1990). Mechanochemical coupling in actomyosin energy transduction studied by *in vitro* movment assay. J. Mol. Biol..

[44] Mitsui T (2002). A supplement to the theory of muscle contraction. Bussei-Kenkyu.

[45] Nothnagel EA, Webb WW (1982). Hydrodynamic models of viscous coupling between motile myosin and endoplasm in Characean algae. J. Cell Biol..

[46] Rivolta MN, Urrutia R, Kachar B (1995). A soluble motor from the alga Nitella supports fast movement of actin filament *in vitro*. Biochim. Biophys. Acta.

[47] Higashi-Fujime S, Ishikawa R, Iwasawa H, Kagami O, Kurimoto E, Kohama K, Hozumi T (1995). The fastest actin-based motor protein from the green algae, Chara, and its distinct mode of interaction with actin. FEBS Lett..

[48] Mitsui T, Ohshima H (2005). Proposed model for the flagellar rotary motor. Colloid and Surfaces B: Biointerfaces.

[49] Mitsui T, Ohshima H (1988). A self-induced translation model of myosin head motion in contracting muscle I. Foece-velocity relation and energy liberation. J. Musc. Res. Cell Motility.

[50] Woledge RC, Curtin NA, Homsher E, Woledge RC, Curtin NA, Homsher E (1985). Chapter 4 Heaat production and chemical change. Energetic Aspects of Muscle Contraction.

